# Biomimetic Microfluidic Platforms for the Assessment of Breast Cancer Metastasis

**DOI:** 10.3389/fbioe.2021.633671

**Published:** 2021-03-11

**Authors:** Indira Sigdel, Niraj Gupta, Fairuz Faizee, Vishwa M. Khare, Amit K. Tiwari, Yuan Tang

**Affiliations:** ^1^Biofluidics Laboratory, Department of Bioengineering, College of Engineering, University of Toledo, Toledo, OH, United States; ^2^Eurofins Lancaster Laboratories, Philadelphia, PA, United States; ^3^Department of Pharmacology and Experimental Therapeutics, College of Pharmacy & Pharmaceutical Sciences, University of Toledo, Toledo, OH, United States

**Keywords:** microfluidics, tumor microenvironment (TEM), invasion, intravasation, extravasation, organotropism, metastatic niche, breast cancer metastasis

## Abstract

Of around half a million women dying of breast cancer each year, more than 90% die due to metastasis. Models necessary to understand the metastatic process, particularly breast cancer cell extravasation and colonization, are currently limited and urgently needed to develop therapeutic interventions necessary to prevent breast cancer metastasis. Microfluidic approaches aim to reconstitute functional units of organs that cannot be modeled easily in traditional cell culture or animal studies by reproducing vascular networks and parenchyma on a chip in a three-dimensional, physiologically relevant *in vitro* system. In recent years, microfluidics models utilizing innovative biomaterials and micro-engineering technologies have shown great potential in our effort of mechanistic understanding of the breast cancer metastasis cascade by providing 3D constructs that can mimic *in vivo* cellular microenvironment and the ability to visualize and monitor cellular interactions in real-time. In this review, we will provide readers with a detailed discussion on the application of the most up-to-date, state-of-the-art microfluidics-based breast cancer models, with a special focus on their application in the engineering approaches to recapitulate the metastasis process, including invasion, intravasation, extravasation, breast cancer metastasis organotropism, and metastasis niche formation.

## Introduction

Breast cancer is the second leading cause of cancer death in women (Centers for Disease control and Prevention, 2018). Metastasis is a key event of cancer progression and the primary cause of mortality in breast cancer patients (Jin and Mu, 2015). Breast cancer cells may metastasize through axillary lymph nodes or systemic circulation with the latter one being the dominant route for cancer dissemination (Ullah et al., 2018). The formation of metastasis is a multistage process that requires breast cancer cells to survive several rate-limiting steps including escaping from the primary site, survival in the circulation, seeding at distant sites, and proliferation (Joyce and Pollard, 2009). Despite the clinical importance of breast cancer metastases, research has largely focused on the oncogenic transformations leading to the development of primary tumors and much remains to be learned about the metastatic process. Traditionally, preclinical studies of breast cancer metastasis rely heavily on murine models, though validation efforts are rare, even in the era of targeted therapy where understanding the genetic signatures of tumors under study is critical (Rashid and Takabe, 2015). In recent years, concerns regarding genomic and phenotypic correspondence between human and murine models and their relevance to human breast cancers have been expressed by the scientific community (Mollard et al., 2011; Gengenbacher et al., 2017). For this reason, xenograft models that are grown in immunocompromised mice employing human patient-derived breast cancer samples (PDX) have emerged as a powerful tool for understanding tumor characteristics. PDX models of human breast cancer maintain a relatively high level of genomic, transcriptional, and phenotypic fidelity compared to the original patient tumor which allows for capturing of the genetic complexity of human breast cancers and is superior to genetically engineered mice (Holen et al., 2017) and thus, is recommended as a tool to mimic human clinical trials (Pompili et al., 2016). Nevertheless, despite all these advantages, PDX models did not entirely overcome all the limitations associated with classical xenograft models (Sakamoto et al., 2015). For example, PDX models do not allow for genetic manipulations of the transplanted tumor. More importantly, any studies which require an intact immune system such as breast cancer immunotherapy studies are almost impossible to conduct in PDX models since the recipient mice are immunocompromised. Overall, *in vivo* models are expensive and require skilled personnel while still cannot model all aspects of the interaction and crosstalk between human cancer cells, human immune cells, and human tissue. Moreover, strictly regulated, reproducible parametric studies are difficult to perform.

In contrast to *in vivo* studies, *in vitro* models are cost-effective means for pre-clinical studies of breast cancer metastasis. Transwell-based assays have been widely used to investigate breast cancer metastasis, especially migration, and invasion (Sontheimer-Phelps et al., 2019). 3D *in vitro* tumor models, such as the spheroid hanging drop methods, can incorporate multiple cell types and thus better recapitulates cell–cell/cell–ECM interactions in primary breast tumors. However, these static models do not account for transport across the vascular endothelium and do not reproduce the complex network structure, fluid shear, hydrostatic pressure, and tissue deformation observed in the *in vivo* tumor microenvironment (TME). Furthermore, they rely exclusively on diffusion and thus are not suitable for the study of drug molecule-breast tumor interaction or the recruitment of circulating immune cells. Due to this limitation, and given the complexity of existing *in vivo* models, recent research has shifted focus to the development of microfluidics-based devices to study the stepwise metastasis of breast cancers (Peela et al., 2017; Bray et al., 2019; Sontheimer-Phelps et al., 2019; Trujillo-de Santiago et al., 2019; Coughlin and Kamm, 2020). These *in vitro* “organ-on-chip” models, although unable to fully replicate the *in vivo* situation, can overcome some of these limitations by using human cells throughout and providing highly controllable environments where single culture parameters can be modified. These devices, fabricated of PDMS, glass, or other biocompatible, optically clear thermoplastic materials to allow for real-time monitoring, are structured with channels/chambers to facilitate the recapitulation of a physiologically relevant breast TME featuring complex multicellular structures in a well-controlled manner. In this review, we will provide readers with a detailed discussion on the most up-to-date, state-of-the-art microfluidics-based breast cancer models, with a special focus on their application in the engineering approaches to recapitulate the metastasis process.

## The Breast Tumor Microenvironment

The normal breast microenvironment is composed of extracellular matrix (ECM) and stromal cells (e.g., endothelial cells, immune cells, fibroblasts, and adipocytes) that are embedded within it. Similar to normal tissue microenvironment, breast tumor cells in the TME are embedded in the ECM surrounded by blood vessels to supply nutrition and oxygen (Spill et al., 2016). The critical role of breast TME in tumor growth and therapeutic response has been increasingly recognized, particularly as it relates to breast cancer metastatic progression. In recent years, various aspects of breast tumor TME has been extensively reviewed (Di Virgilio et al., 2018; Hamidi and Ivaska, 2018; Lim et al., 2018; Spranger and Gajewski, 2018; Costard et al., 2021), however, there is still much to be learned in our effort to develop novel therapies targeting the TME. Early stage microfluidic devices usually employ several parallel straight microchannels for easy access and imaging, therefore, is capable of studying cell migration (Wong et al., 2012), however, these devices have not been optimized for studying sophisticated interactions between tumor, endothelium, stroma, immune cells, chemokines, or cancer stem cells (Sridharan et al., 2019) observed in the complex TME. In recent years, more advanced “breast cancer metastasis-on-chip” type devices featuring dedicated tumor compartment for breast cancer growth and endothelial cell covered microchannels for mimicking tumor vasculature have emerged for the study of the TME leading to metastasis. Compartmentalized design enables multi-cellular co-culture; whereas a complex microchannel network allows for independent fluidic perfusion or sampling from individual compartments within the same device which then facilitates parametric control of microenvironmental factors (Esch et al., 2015).

**FIGURE 1:**
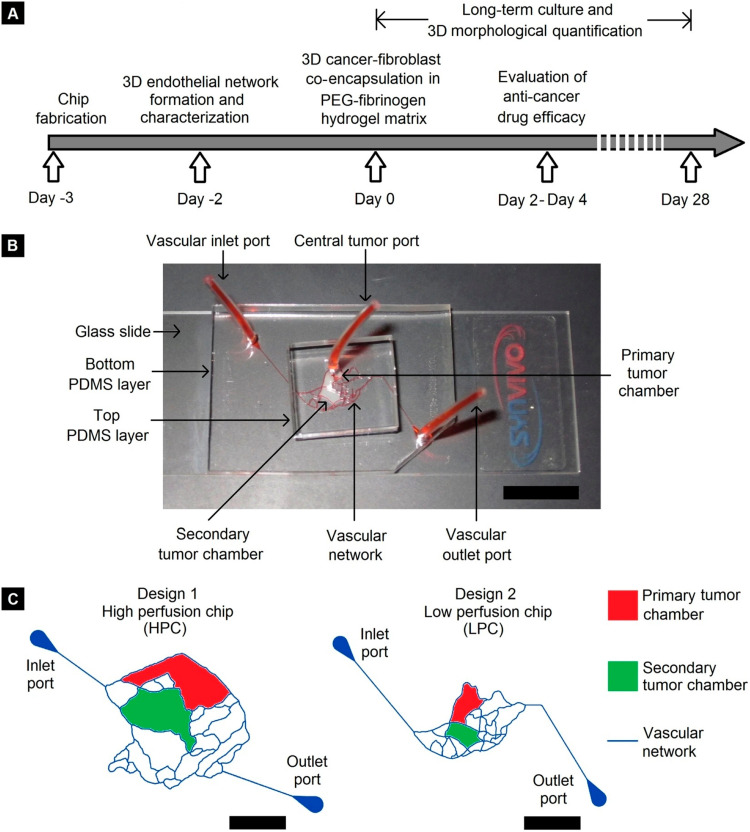
Microfluidic device designed by Pradhan et al. (2018). **(A)** Timeline of device fabrication, endothelium formation, 3D breast tumor-fibroblast co-culture, and long-term characterization. **(B)** Eosin Y perfusion showing the vascular channels and tumor compartments (Scale bar = 1 cm). **(C)** Schematic representation of the microchannel network, primary and secondary tumor compartments (two different designs) (Scale bar = 5 mm). Reproduced with permission (Pradhan et al., 2018). Licensed under CC BY-NC 4.0. https://creativecommons.org/licenses/by-nc/4.0/.

More and more researchers are now applying this “biomimetic” approach to achieve pathophysiological fidelity and clinical relevance. These devices usually feature endothelial cell covered microchannel network that is recreated from *in vivo* vasculature geometry, dedicated tissue compartments filled with ECM materials for 3D breast cancer-stroma co-culture, and optical transparency which offers real-time visualization. A good example of such an application is provided in the work of Pradhan et al. (2018) where multiple levels of physiological complexity were incorporated into a PDMS-based microfluidic platform, as illustrated in Figure 1. This breast cancer microenvironment on-chip features a dedicated primary tumor site (red) for breast tumor growth and a secondary tumor site (green) for metastasis. The tumor compartments were surrounded by endothelial cell-covered vascular channels. Metastatic (MDA-MB-231) or non-metastatic (MCF-7) breast cancer cells and stromal fibroblasts were grown within a PEG-fibrinogen hydrogel matrix in the primary tumor compartment (red). The geometric features of the vascular channels were directly replicated from the pathophysiological architecture of breast tumor vasculature obtained by *in vivo* imaging and therefore facilitated the formation of mature, lumenized endothelium when cultured under physiological flow shear conditions. Due to the flow-based design, the authors achieved long term cancer-stromal-endothelial co-culture (at least 28 days) within the microfluidic chip, enabling the investigation of cancer-stromal-endothelial interaction as well as morphogenic tumor metastasis over time. This microfluidic platform highlights the role of a fluidic, biomimetic vascular network in recapitulating the breast TME.

Due to the flow-based design, microfluidics-based platforms are inherently suitable for breast cancer metastasis study. Microfluidics offers one of the only means for mimicking vascular conditions and controlling specific factors such as flow rate experimentally (Simpson et al., 2019). Through regulation of the flow perfusion, microfluidic assays enable quantitative analysis of diverse biological processes in high-resolution. One such example can be seen in the works of Tang et al. (2017). To better understand the impact of different types of breast cancers on tumor vasculature, Tang et al. (2017) developed a microfluidics-based breast TME recapitulating circulation, intravasation, and drug delivery to the tumors across the interstitial space. The perfused microfluidic platform, fabricated using PDMS-based soft lithography, contains primary human breast tumor-associated endothelial cells forming a lumen in the microchannel network in communication with breast tumor suspended in Matrigel in a tumor compartment. The authors showed that aggressive subtypes of breast tumors led to leakier endothelium in the adjacent tumor vasculature than less aggressive subtypes through precise quantification of endothelial permeability, which was measured with real-time fluorescence imaging to the microfluidic chip while small-molecule fluorescent tracers transport across the endothelium. It is worth noting that the tumor vasculature hyperpermeability is a response to the presence of either tumor cells or tumor cell-conditioned medium. Via on-chip immunostaining assays, this phenotypical change in the endothelium was later attributed to the increased disruption of both tight and adherens junctions by the metastatic breast cancer, which has also been observed *in vivo*, indicating the capability of the microfluidics-based assays in recapitulating critical events during breast cancer metastasis.

As illustrated by these representative studies, the microfluidics-based metastasis-on-chip approach could expand our fundamental knowledge of breast TME and enable a more accurate *in vitro* representation of breast cancer metastasis processes. In recent years, a myriad of studies has used similar microengineering principles to investigate the molecular mechanisms of breast cancer cell invasion (Gioiella et al., 2016; Blaha et al., 2017; Truong et al., 2019; Yankaskas et al., 2019), intravasation (Cui et al., 2017; Nagaraju et al., 2018; Shirure et al., 2018), extravasation (Chen M. B. et al., 2016; Chen et al., 2017; Song et al., 2018; Boussommier-Calleja et al., 2019), breast cancer metastasis organotropism and metastasis niche formation (Bersini et al., 2014; Wheeler et al., 2014; Jeon et al., 2015; Clark et al., 2016b; Xu et al., 2016; Narkhede et al., 2017; Shumakovich et al., 2017; Marturano-Kruik et al., 2018; Mei et al., 2019; Oliver et al., 2019; Kim et al., 2020; Ribeiro et al., 2020; Tian et al., 2020).

## Invasion

Breast cancer invasion is one of the earliest events during metastasis. Changes in the primary TME are the major reason for breast cancer matrix invasion (Cox and Erler, 2011). A variety of biochemical (e.g., excess release of cytokines, chemokines) and biophysical changes in the tumor ECM (e.g., basement membrane breakdown, ECM remodeling) enhance the motility of the cancer cell which expedites its spread and eventually leads to the intravasation of cancer cells into neighboring blood vessels (Friedl and Alexander, 2011). Also, under the influence of breast cancer cells, the stromal tissue undergoes excessive remodeling resulting in marked phenotypical and structural changes leading to altered growth factor expression, and cytokine/chemokine release (Friedl and Wolf, 2003). For example, it has been found that stromal cells such as cancer-associated fibroblasts (CAFs), tumor-associated macrophages (TAMs), and adipose cells play a critical role in breast cancer invasion through secretion of numerous biochemical stimuli (Khamis et al., 2012). PDMS-based microfluidic assays, fabricated with soft lithography, have been widely used to investigate the pathophysiology/pathobiology of breast cancer invasion (Peela et al., 2017). For example, the Konstantopoulos group engineered a Microfluidic Assay for quantification of Cell Invasion (MAqCI) where the abundance and proliferative index of migratory breast cancer cells were characterized to predict the metastatic potential of breast cancer cells isolated from commercially available cell lines (MDA-MB-231) and patient-derived xenografts (PDX) (Yankaskas et al., 2019). The device comprising of parallel Y-shaped microchannels was designed and the location information of the migrated breast cancer cells was used as an index of motility. By comparing to breast-cancer cell lines of known metastatic behavior, the authors show that the relative abundance of migratory cells is positively correlated with their metastatic potential (Wan and Kamm, 2019). With a narrow range of threshold percentage (7–9%) and experiment duration (12–14 h), the total accuracy of MAqCI was maximized. The authors obtained an optimal accuracy (96%) with high sensitivity (89%) and specificity (100%), which corresponds to a 100% positive prediction and a 96% negative prediction. The author’s results suggest that highly motile breast cancer cells isolated by the device exhibited similar tumorigenic potential *in vitro* but markedly increased metastatic tendency *in vivo* (propensity Further study using RNA sequencing analysis revealed that these highly motile cells has a high expression of motility and survival related genes) (Yankaskas et al., 2019). The approach therefore has the potential to be developed into a metastasis prediction assay for patients with breast cancer.

Before metastasis, it is well known that breast tumor modifies its local ECM to prepare for its progression (Neri et al., 2016; Sreepadmanabh and Toley, 2018), yet the detailed process of tumor stroma activation remains poorly understood. Microfluidic microengineering techniques provide a versatile tool to recreate the breast TME and may prove suitable for the study of ECM activation. One such example is the microfluidic assay developed by Gioiella et al. (2016) where both epithelial breast cancer cells (MCF7) and stroma (fibroblast-assembled ECM) were co-cultured in a single device. The tumor (370 μm × 780 μm × 300 μm) and stromal (1200 μm × 1370 μm × 300 μm) chambers were separated by a barrier region (arrays of regularly spaced posts of dimensions 120 μm × 120 μm × 300 μm) created in the PDMS which provides spatial control and easy tracking of their physical interactions leading to breast tumor ECM remodeling (Gioiella et al., 2016). The two chambers were individually perfused by dedicated microfluidic channels (200 μm × 4000 μm × 300 μm for tissue and 400 μm × 4000 μm × 300 μm for tumor) for cell loading and perfusion, thus guaranteeing nutrient/oxygen supply and waste removal. The authors claimed that a high degree of similarity, including tissues ECM phenotypic activation, metalloproteinases (MMPs) overexpression, and endogenous collagen network architecture time evolution, was observed utilizing this microfluidic assay when compared with established *in vivo* models. The role of non-malignant cells in breast TME is also critical in metastasis, notably in cell migration (Millet et al., 2019). Truong et al. (2019) established a microfluidics-based 3D-organotypic model to characterize breast tumor invasion that is driven by stroma activation. In this PDMS microfluidic model, breast cancer (SUM-159) and CAFs were co-cultured side-by-side to mimic the breast TME. This spatial arrangement enables cancer-stroma communication while allowing for real-time image acquisition. Live-cell imaging indicates increased cancer cell migration speed while transcriptome analysis further revealed novel molecular targets associated with breast cancer invasion. Interestingly, this enhancing effect of breast cancer cell migration was also observed in endothelial cells. By studying breast tumor invasion within a 3D collagen matrix in a microfluidic chamber, Blaha et al. (2017) revealed a significantly enhanced matrix invasion of metastatic breast cancer cells (MDA-MB-231) with the presence of endothelial cells (HUVEC) co-culture. Overall, the dynamic interactions between breast cancer cells and stromal cells in the tumor ECM are essential for cancer metastasis. Recapitulating these interactions with high-fidelity is a key to understanding the molecular regulators and mechanisms of this process. In this regard, this microfluidics-based *in vitro* models may prove invaluable in the effort of recreating accurate breast TME and understanding the various biochemical processes and signaling pathways involved in breast cancer ECM invasion.

## Intravasation

Intravasation is the process where cancer cells enter the blood circulation via the endothelial lining of blood or lymph vessels (Chiang et al., 2016). Breast cancer cells may metastasize through axillary lymph nodes or systemic circulation with the hematogenous route being the dominant route for dissemination (Ullah et al., 2018). Breast cancer intravasation can be active or passive. During active intravasation, chemokine gradient in the TME drives breast cancer cell migration toward the blood or lymphatic vessels (van Zijl et al., 2011). Meanwhile, breast cancer cells can passively shed from the primary tumor mass and enter the circulation when the tumor grows into the surrounding vessels (Bockhorn et al., 2007). A breast cancer cell can intravasate by paracellular (through endothelial junctions, e.g., adherens junction, tight junction) or transcellular transport (through the body of the endothelial cell) once reaching the endothelial barrier. Traditional assays (e.g., Boyden chamber/transwell, wound assay, and others) have been widely used to study cancer cell transendothelial migration in response to chemotactic gradients, where organ-specific endothelial cells can be pre-cultured on a porous membrane and allowed to grow before cancer cell seeding (Li and Zhu, 1999; Zhang et al., 2012). However, shear forces from the flow are required to maintain a functioning endothelium whereas these *in vitro* models do not provide flow control over the local environment, not to mention complex interactions cannot be accurately analyzed, and imaging is limited. To mimic the fluid shear and address the need for precise control of chemotactic gradient over time, Cui et al. (2017) developed a microfluidic device by photolithography of the transparent, biocompatible SU-8 photoresist. The developed multilayer microfluidic device consists of an endothelial cell covered porous membrane sandwiched by two flow layers. The top fluidic layer is designed for seeding breast cancer cells and to provide flow control. Underneath the endothelial cell layer, the bottom fluidic layer contains multiple independent microchambers so that the transmigrated breast cancer cells can be selectively collected. The authors quantified breast cancer cell transmigration in response to different shear stress levels (2.5 and 10 dyn/cm^2^; corresponding to 5 and 20 μl/min flow rate respectively) as well as a chemotactic gradient (100 ng/ml of the chemokine CXCL12). Through investigation of the migrated breast cancer cells, the author suggests that the breast cancer cell nuclear palladin expression is a critical indicator of the transmigration capability, as consistently higher palladin expression was identified over the nuclear region of the migrating cells compared to that of the non-migrating cells.

Recreating tumor vasculature with confluent endothelial cells is the key to establishing a physiologically relevant *in vitro* breast cancer intravasation model. In most models, vascularization was achieved by embedding endothelial cells in prefabricated microfluidic channels coated with ECM materials (e.g., fibrin, fibronectin, collagen, and gelatin) though devices featuring self-assembled capillary networks are also emerging (Peela et al., 2017; Coughlin and Kamm, 2020). For example, in a microfluidic model that is created by Nagaraju et al. (2018) to study breast cancer cell invasion and intravasation, tumor (MDA-MB-231), stroma (fibroblast), and endothelium (HUVEC) were cultured in three independent yet interconnected compartments that are filled with different types of ECM materials. Fibroblast housed in the middle compartment was grown within acellular collagen; MDA-MB-231 and HUVEC were cultured in the two side compartments that are filled with collagen gel and fibrin gel respectively. This arrangement facilitates the reciprocal interaction between tumor, stroma, and endothelium that is observed in the *in vivo* tumor TME and is the key to the simultaneous assessment of invasion and intravasation. Significantly enhanced MDA-MB-231 invasion into the stromal region was observed when HUVECs are present in the vascular compartment, whereas MDA-MB-231 intravasation into the vascular compartment only happens with the presence of HUVECs suggesting that the intravasation process is driven by an endothelial cell generated chemokine gradient.

Self-assembled endothelialized capillary networks have been successfully established in microfluidic devices for the on-chip organoid culture of breast cancer cells. In a model developed by Shirure et al. (2018), a 3D perfusable blood vessel network was created using a 1:2 mixture of human endothelial cells and fibroblast in ECM containing bovine fibrinogen (10 mg/ml) and bovine plasma thrombin (2 U/ml). The cells begin to self-assemble into a vascular network by day 1 and a full-formed network is present by day 7 in the vascular compartment of a PDMS-based microfluidic device (Shirure et al., 2018). Upon continuous perfusion of culture media, the authors successfully maintained breast cancer organoid culture for 22 days. Interestingly, the incidence of tumor intravasation was rare (<5 cells) considering the relatively high number of tumor cells present in the vascular compartment (on the order of hundreds). Meanwhile, increasing the endothelium permeability by VEGF or thrombin treatment did not significantly increase the rate of intravasation. Considering the low incidence of intravasation, the possibility to support the tumor organoids culture for 22 days in this device represents a practical advantage in studying intravasation when compared with the conventional organoid culture which usually has limited nutrient supply and life span.

Overall, microfluidics-based models have greatly improved our understanding of the mechanisms of breast cancer intravasation, especially regarding the hematogenous spread. However, the recapitulation of breast tumor intravasation via the lymphatic route has yet to be explored within a microfluidic model (Peela et al., 2017). Moreover, almost all the studies have focused on paracellular intravasation with a particular interest in the disruption of endothelial junctions during breast cancer intravasation whereas the transcellular intravasation phenomenon has not been extensively studied.

## Extravasation

A final step of breast cancer cell metastasis is extravasation out of a blood vessel at the distant site, a process that is fundamentally different from intravasation but similar to the leukocyte extravasation process during inflammation (Strell and Entschladen, 2008; Reymond et al., 2013) where circulating tumor cells (CTCs) under blood flow shear exhibit a leukocyte-like “rolling” behavior (transient adhesive contact) before firmly adhere to the endothelium at the distant organ (Friedl and Wolf, 2003; Azevedo et al., 2015). This process is mediated by interactions between upregulated endothelial cell adhesion molecules and ligands expressed on the surface of the CTCs that recognize them. Receptors on the CTC surface, such as glycoproteins sialyl Lewis-a/x (Rho et al., 2014) and the selectins (e.g., *P*-, *E*-, and L-selectin) on the endothelial cell surface regulates CTC initial capture and rolling (Burdick et al., 2012), whereas firm adhesion and transmigration are mediated by a combination of integrins/immunoglobulins (e.g., ICAM-1, VCAM-1, L1-CAM) and chemoattractants in the tissue (Strell and Entschladen, 2008; Bendas and Borsig, 2012; Barbazan et al., 2017).

**FIGURE 2:**
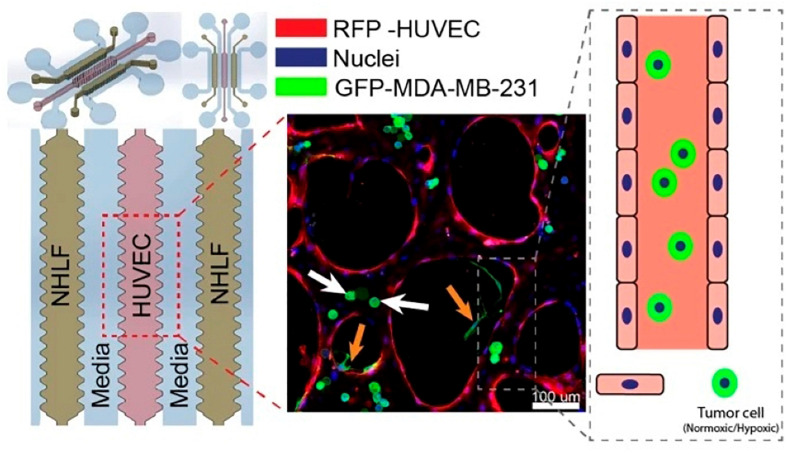
A microfluidics design by Song et al. (2018) for the investigation of the role of HIF-1α in breast cancer extravasation. From the left: media channel – NHLF channel (suspended human lung fibroblast) – media channel – central gel region (suspended HUVECs) – media channel – NHLF channel (suspended human lung fibroblast) – media channel. A representative fluorescence image of co-cultured cells was shown in the middle. Reproduced with permission (Song et al., 2018). Licensed under CC BY-NC 4.0. https://creativecommons.org/licenses/by-nc/4.0/.

Kamm’s group carefully examined the role of β1 Integrin, a critical factor involved in neutrophil locomotion and extravasation (Werr et al., 1998), of circulating breast cancer cell extravasation using a microfluidic assay (Chen M. B. et al., 2016). In this study, Chen M. B. et al. (2016) designed a microfluidic device composed of three hydrogel regions separated by media channels which were then seeded with HUVECs, forming a monolayer, and different cancer cell types, including breast cancer cell MDA-MB-231. Their results showed that expression of β1 integrin was necessary for breast cancer cells to stabilize protrusions that insert in between endothelial cells and contact the underlying basement membrane. Knockdown of β1 integrin in cancer cells resulted in a decrease in their ability to form stable protrusions and extravasate, leading to decreased *trans*-endothelial migration. Due to the tight cross-linking structures present in the basement membrane, the authors concluded that it is unlikely for breast cancer cells to pass through without degrading the ECM. Therefore, further studies may be needed to elucidate the role of β1 integrin in mediating the production of metalloproteinases (an enzyme that can degrade ECM). It is also worth noting that the integrin profile of MDA-MB-231 has been previously characterized and the expression of a variety of αα-subunits was discovered (Haidari et al., 2012); however, their role in extravasation was unclear. For this reason, the authors also performed knockdown experiments of various α-subunits and showed that α3 and α6 were involved in MDA-MB-231 extravasation while αv and α5 were not. Utilizing the same microfluidic platform (Figure 2), Song et al. (2018) from the Kamm group also investigated the extravasation potential of hypoxic human breast epithelial and cancer cell lines and confirmed the critical role of HIF-1α in the extravasation process. Hypoxia-inducible factors (HIFs) are a family of transcription factors that regulate the expression of certain genes that respond to reductions in oxygen concentration. These heterodimeric complexes are made up of an oxygen sensing α-subunit whose expression increases under hypoxia and a constitutively expressed β-subunit (Harris, 2002). Cellular processes controlled by HIFs have been associated with cancer development with HIF-1α being of particular interest since it has been described to promote an aggressive cancer phenotype, regulate the expression of various chemokines and cytokines (Mojsilovic-Petrovic et al., 2007; Tian et al., 2014), and control many important steps of the metastasis cascade (Jin et al., 2012; Semenza, 2012). The authors studied the contribution of hypoxia and HIF-1α to the extravasation potential of MCF-7, MCF10A, and MDA-MB-231 breast cancer cells. The microfluidic device contained three hydrogel regions with endothelial cells (HUVEC) in the center and fibroblast in the side regions. Separating the hydrogel regions were two media channels. HUVECs self-organize into microvascular networks within 4–5 days in the hydrogel, after which tumor cells can be introduced and extravasation events tracked via microscopy over 72 h (Chen et al., 2017). A pressure gradient (5.2 mm H_2_O) was applied across the microvasculature and each breast cell line, either pretreated under hypoxic (1% O_2_, 5% CO_2_, 37°C) or normoxic (21% O_2_, 5% CO_2_, 37°C) conditions were suspended in the media channels for 5 days. Control cells were cultured simultaneously in a normoxic incubator. Cells that were either adherent or physically trapped inside the endothelium were imaged and analyzed. The authors also explored the impact of pre-treatment with hypoxia on the extravasation rate of the three breast cell lines. It was found that the extravasation rates on average (33.28 ± 2.49%, 50.45 ± 6.15%, and 66.41 ± 4.45% for MDA-MB-231, MCF-7, and MCF-10A respectively, a 1.5–3.5-fold increase compared to normoxic cells) were significantly higher in cells pretreated under hypoxic conditions versus those pretreated under normal conditions. Furthermore, cells pretreated under hypoxic conditions with siRNA knockdown of HIF-1α showed a significant reduction in extravasation rates compared to their wildtype counterparts. Notably, the extravasation rate was similar to that of the wild-type cells cultured in normoxic conditions, indicating the critical role of HIF-1α.

The inflammatory response correlates with breast cancer metastatic potential, including enhanced extravasation (St Hill, 2011; Geng et al., 2013). For example, *in vivo* analysis of CTC spread revealed preferential dissemination of breast tumor cells to endothelium within organs that express high levels of the CXC- chemokine ligand 12 (CXCL12) (Sun et al., 2014). Pro-inflammatory cytokines such as TNF-α, IL-1 can upregulate several adhesion molecules on endothelial cells and promote metastatic cell adhesion and transmigration (Mannel et al., 1994; Stoelcker et al., 1995; Eichbaum et al., 2011). In the liver, breast cancer cells can initiate an inflammatory cascade, which increases adhesiveness to liver sinusoidal endothelium cells (Khatib et al., 2005). Expression of proinflammatory cytokine IL-1 by primary breast cancer cells strongly correlates with bone metastasis (Tulotta and Ottewell, 2018). The consistent high *E*-selectin expression level in the bone micro-vessels (Schweitzer et al., 1996; Barthel et al., 2007) might be the reason for the high incidents of breast cancer bone metastasis. In a rodent lung lipopolysaccharide inflammation model, breast cancer lung metastasis was significantly increased via *E*-selectin upregulation in the lung endothelium (Jiang et al., 2014) probably through the production of pro-inflammatory cytokine TNF-α (Eichbaum et al., 2011), which can upregulate several adhesion molecules on endothelial cells and promote metastatic cell adhesion and migration (Mannel et al., 1994; Stoelcker et al., 1995), including *E*-selectin, *P*-selectin, ICAM-1, and VCAM-1. In the brain, breast cancer cell extravasation across the blood-brain barrier (BBB) can be significantly exacerbated due to the inflammatory mediator COX-2 secretion by the brain capillary endothelial cells (Lee et al., 2011). Increased serum level of IL-1 and TNF-α has been shown by several different studies to be correlated with increased metastatic potential (Jin et al., 1997; Sheen-Chen et al., 1997; Chavey et al., 2007; Rafiq et al., 2007; Goldberg and Schwertfeger, 2010; Papadopoulou et al., 2010; Esquivel-Velazquez et al., 2015).

Microfluidic models enabled the precise control and manipulation of a biological target and analysis of functional outcomes of target modulation. For this reason, various microfluidics-based transendothelial migration assays have been developed to elucidate the exact regulation mechanism of breast cancer extravasation during inflammation. For example, Song et al. (2009) from Takayama’s group successfully reproduced the CXCL12 mediated breast cancer cell preferential adhesion to endothelial cells *in vitro* using a multi-layered microfluidic device. Their results further revealed that the recruitment of MDA-MB-231 breast cancer cells is mediated by the CXCR4 receptor on endothelial cells and is independent of the CXCL12 level on the breast cancer cells, suggesting the potential of CXCL12-CXCR4 inhibition as a valid therapeutic target for preventing metastasis. Using their hydrogel model, Kamm’s group investigated the role of immune cells, such as macrophages and monocytes in the TME and characterized the properties of endothelium leading to extravasation (Zervantonakis et al., 2012; Jeon et al., 2013; Boussommier-Calleja et al., 2019). Their results suggest a non-contact dependent reduction effect of monocytes on breast cancer cell extravasation, which quickly vanished once monocytes transmigrate across endothelial cells and become macrophage-like. These findings, obtained in microfluidic devices, not only replicated phenomena observed *in vivo* but also discovered previously undefined roles of the inflammatory microenvironment in vascular endothelium and breast cancer cell extravasation.

Other than the leukocyte model of extravasation, CTCs can also directly arrest within small diameter blood vessels due to size restriction, a process termed mechanical trapping, resulting in enhanced receptor-ligand interaction and a higher chance of extravasation (Kienast et al., 2010). Compared to the well-characterized leukocyte model, studies aiming at understanding the mechanical trapping process is lacking. It is believed that size restriction combined with firm adhesion is the dominant route for extravasation (Coughlin and Kamm, 2020), however, further studies are required to elucidate the relative contribution of the two routes in governing breast cancer extravasation.

## Metastasis Organotropism and Metastatic Niche

**FIGURE 3:**
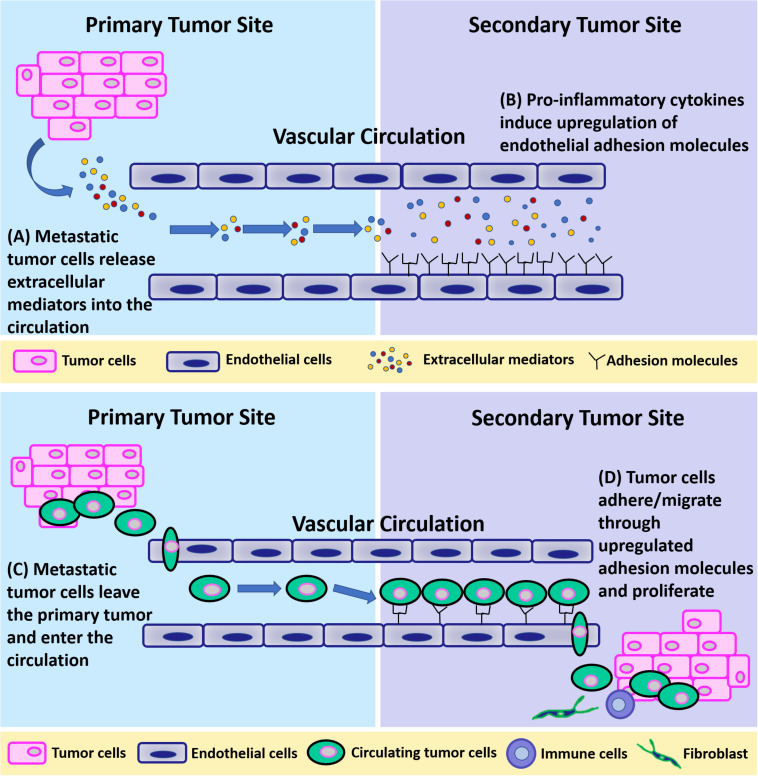
A schematic of breast tumor metastasis. **(A)** Metastatic tumor cells release extracellular mediators into the circulation. **(B)** Pro-inflammatory cytokines released from primary tumors induce upregulation of endothelial adhesion molecules at the secondary site. **(C)** Metastatic tumor cells leave the primary tumor and enter the circulation. **(D)** Tumor cells adhere to and migrate through endothelial cells through upregulated adhesion molecules at the secondary site and proliferate.

Circulating breast cancer cells can extravasate at any distant organ (Cifuentes and Pickren, 1979; Lee, 1983) but primarily at the bone, lung, regional lymph nodes, liver, and brain (Xu et al., 2011), with bone being the most preferred site of metastasis (75% of stage IV breast cancer patients develop skeletal metastases) (Brook et al., 2018; Tulotta and Ottewell, 2018). This non-random, organ-specific metastasis behavior is known as “organotropism” (Gao et al., 2019). Clinical research has identified several molecular mediators and genes whose expression specifically promotes extravasation and metastatic colonization of breast cancer to bone, lung, and brain (Lorusso and Ruegg, 2012). In general, it is believed that breast cancer metastatic organotropism is a result of multiple different factors including blood flow patterns, breast cancer subtype, host microenvironment, and cancer-host cell interaction (Chen et al., 2018). This pattern of metastases was explained by the so-called “seeds and soil” hypothesis by Paget (Paget, 1989; Lu and Kang, 2007), in which the unique properties of particular tumor cells (seeds) and the different characteristics of each organ microenvironment (soil) collectively determine the organ preference of metastasis (Fidler, 1989). To get effective homing and finally colonize within distant organs, circulating breast cancer cells need a “fertile” microenvironment. Primary breast tumors can induce the formation of a pro-survival microenvironment, termed pre-metastatic niche (PMN), in distant organs before their arrival. PMN consists of thin vasculature predominantly made up of endothelial cells and is regulated by tumor cell-secreted factors and tumor shed extracellular vesicles (EVs) such as exosomes, to initiate non-resident cell recruitment, and host cell alternations (Liu and Cao, 2016; Peinado et al., 2017). Taken together, it is highly possible that metastatic breast tumor at the primary site can remotely induce an inflammatory microenvironment at certain distant organs for circulating breast cancer cells to adhere and extravasate by releasing EVs containing proinflammatory cytokines. Metastatic cells first loosely adhere to the vasculature and make initial cell-cell connections through upregulated cell adhesion molecules including selectins (*E*-selectin, *P*-selectin), and *N*-cadherin (Mine et al., 2003; Draffin et al., 2004; Kobayashi et al., 2007; St Hill, 2011; Bendas and Borsig, 2012; Mitchell and King, 2014). Then, firm adhesions are formed between metastatic cells and endothelium, which are mediated by integrins (ICAM-1, VCAM-1), mucins, and CD44 (Khatib et al., 2005; Rahn et al., 2005; Schlesinger and Bendas, 2015). Lastly, adhered cancer cells migrate between endothelial cells through damaged cellular junctions (adherens junctions, tight junctions). This process is illustrated in Figure 3.

## The Bone Niche

There are several *in vivo* and *ex vivo* models developed to study breast cancer metastasis organotropism (Naumov et al., 1999; Al-Mehdi et al., 2000; Kuperwasser et al., 2005; Goldstein et al., 2010; Stoletov et al., 2010), with most studies focus on the bone metastasis employing intravenous, intracardiac or direct skeletal injection of breast cancer cells in murine models. To understand bone metastasis, it is crucial to understand the breast cancer PMN in the bone marrow. The bone marrow microenvironment consists of stem cells [both hematopoietic (HSCs) and mesenchymal (MSCs)], osteoblasts, osteoclasts, and adipocytes embedded in a collagen-rich mineralized ECM (Narkhede et al., 2017). The formation of the bone PMN is predominantly regulated by the HSCs secreted vascular endothelial growth factor (VEGF) (Saxena and Christofori, 2013) and osteoblasts secreted CXCL12 (Sun et al., 2010) which, as explained previously, plays a key role in extravasation.

To understand the events leading to breast cancer bone metastasis, microfluidics approaches have been developed to mimic the bone niche. Since inflammatory microenvironment correlates with increased breast cancer metastatic potential, it is reasonable to hypothesize those pro-inflammatory chemokines which are constitutively expressed at the bone niche direct the extravasation of the CTCs to these organs (Ben-Baruch, 2003). To test this, a 3D microfluidic model was created by the Kamm group to simulate the complex interactions between CTCs, vascular endothelium and bone tissue to investigate human breast cancer bone metastasis (Bersini et al., 2014). To reconstitute the tissue-tissue interface between the endothelium and bone, Bersini et al. (2014) used HUVECs to form the inner lining of a microfluidic channel that is adjacent to bone cells differentiated from human bone marrow-derived MSCs (hBM-MSCs) in a 3D collagen gel. Breast cancer metastasis was examined using real-time microscopy to track adhesion and extravasation of breast cancer cells that were circulating in the vascular channel and their subsequent migration deeper into the bone matrix. Using immunofluorescent staining, the authors demonstrated the presence of CXCR2 surface receptors on MDA-MB-231 cells. Antibody blocking of CXCR2 reduced MDA-MB-231 extravasation from 77.5 ± 3.7% to 45.8 ± 5.4%, whereas the addition of CXCL5 ligand led to an increase in the extravasation percentage from 37.6 ± 7.3% to 78.3 ± 9.7%. Their results suggest that the chemokine CXCL5 (produced by bone cells) and its receptor CXCR2 (expressed on breast cancer cells) are major signaling mediators that govern the rate of CTC extravasation and the extent of migration, indicating the possibility of CXCL5-CXCR2 inhibition as a therapeutic target. Using a similar microfluidic model, a follow-up study by Jeon et al. (2015) demonstrated the therapeutic potential of targeting A_3_ adenosine receptor in preventing breast cancer bone extravasation. When breast cancer A_3_ adenosine receptor was blocked with antagonist PSB-10 within the microfluidic device, significantly increased cancer cell extravasation (32.4 ± 7.7% vs. 8.2 ± 2.3%) was observed compared to non-treated controls. Interestingly, the authors observed a disparity between the endothelium permeability and the number of extravasated breast cancer cells, which implies that permeability is only one of many factors contributing to extravasation.

The skeleton is a mechanically dynamic tissue under constant modeling and remodeling. Osteocytes stimulate bone formation in the presence of mechanical stimuli, as well as bone degradation in the absence of such stimuli (Klein-Nulend et al., 2012). It is well known that lack of physical activity can cause bone loss and fractures. Clinical and *in vivo* studies suggest that exercise can reduce the rate of adverse events and tumor formation in bone (Lynch et al., 2013; Sheill et al., 2018), however, the role of mechanical loading in breast cancer bone metastasis is not clear. For this reason, Mei et al. (2019) investigated the effect of mechanically stimulating osteocytes on breast cancer bone metastasis using a microfluidic device composed of two channels: one containing a 3D lumen of HUVECs and MDA-MB-231 breast cancer cells, and the other containing osteocyte like MLO-Y4 cells. These adjacent channels were separated by a hydrogel barrier region to ensure that the cell signaling between the two channels is via diffusion with no convective mass transfer involved. Oscillatory fluid flow (OFF) was induced in the MLO-Y4 containing channel at physiologically relevant conditions (1 Hz, 1 Pa). Cancer cell extravasation was compared when osteocytes were stimulated with fluid shear stresses (0.8–5 Pa) to unstimulated osteocytes. They discovered that the fraction of breast cancer cells that extravasated (from the lumen channel, across the HUVEC layer, to the osteocyte channel) and the distance traveled by these extravasated cells were significantly reduced under the OFF compared to the static culture condition (36.6 μm extravasation distance, 32.4% extravasation rate vs. 110.3 μm extravasation distance, 102.1% rate). The authors hypothesize that this effect is due to increased Prostaglandin E_2_ (PGE2) secretion, a prostaglandin that is capable of reinforcing endothelial barrier (Birukova et al., 2007), by mechanically stimulated MLOY4 cells (Zhang et al., 2015). Unlike visceral organs, bone is much more rigid. Therefore, soft hydrogels may not be the best candidate for mimicking the stiffness and biochemical composition of the bone microenvironment. This requirement can be addressed by replacing soft hydrogel with the decellularized natural bone matrix as the ECM material. Marturano-Kruik et al. (2018) created a microfluidics-based bone PMN in which endothelial cells were mixed with hBM-MSCs and seeded into a decellularized bone matrix. The authors observed a naturally formed dense vascular network after 1 week of flow perfusion (0.25 μL/min perfusion, leading to 0.03–0.32 mPa shear stress distribution within the vascular network according to author’s CDF simulation) and studied the effect of interstitial fluid flow shear, oxygen gradients, and external forces on breast cancer cell colonization within the PMN. Their results revealed that breast cancer cells colonizing the bone PMN entered an interstitial flow triggered slow-proliferative state, leading to drug resistance. The authors hypothesized that this effect is due to the cancer stem cells adapting to the changed bone PMN microenvironment and escaping targeted therapy.

Overall, microfluidics-based bone PMNs provides a biomimetic vascular construct allowing structured yet dynamic communication between breast CTCs and the unique bone microenvironment (osteocytes, MSCs, endothelial cells, chemokine/cytokine gradients, etc.). Similar approaches for exploring breast cancer lung, liver, or brain metastasis have also been reported in the past 2–3 years, albeit much less compared to the vast majority of bone metastasis models.

## The Lung Niche

The lung is the second most common site of breast cancer metastasis (Jin et al., 2018). The incidence of lung metastasis can be as high as 40% in patients with triple-negative breast cancer (TNBC) compared to about 20% in patients with non-TN breast cancers (Jin et al., 2018). Surprisingly, microfluidics-based models aiming at recreating the lung microenvironment leading to breast cancer lung metastasis have been rare. Kong et al. (2016) established such a system for the assessment of the metastatic potential of various cancers to the lung. The system is composed of a bottom PMDS layer for hosting the organ chamber, a top PDMS layer for hosting the microfluidic channel networks, and a porous membrane separating the two. These three layers were then bonded to a glass substrate for structural support. The organ chamber was coated with collagen I, the most abundant ECM protein found in lung tissue, to mimic the lung ECM. Mixed primary pulmonary tissue cells, isolated from rat lungs, were then seeded into the organ chamber. The pulmonary endothelial barrier was reconstituted with HUVECs covering the microchannels that were pre-coated with Cultrex Basement Membrane Extracts. Breast CTC extravasation was induced by chemokine gradient (CXCL12) and imaged while CTCs, introduced from the vascular channels, transmigrate into the organ chamber. After validating their results using a mouse model of breast cancer lung metastasis (tail vein inoculation; 80, 100, and 100% metastasis rate for MCF7, MDA-MB-231, and ACC-M respectively.), the authors successfully characterized the therapeutic potential of an anti-metastatic reagent AMD3100 where increasing AMD3100 concentration (0–2 μg/ml) correlates with decreasing breast cancer colony area.

Although this biomimetic model was able to provide an accurate representation of the breast cancer lung metastasis cascade, it lacks certain key components for us to understand the underlying mechanisms of the breast cancer lung metastasis organotropism. For example, it has been found that the lung epithelial cells (e.g., SAEC), a component that is not studied in this model, mediate the aggressive phenotype of MDA-MB-231 cells by triggering mesenchymal to epithelial transition (MET) (Furukawa et al., 2015). Further, breast cancer extravasation is for the most part regulated by different endothelial barriers of the host organ. The lung has a vast endothelial surface area, which is essential for the exchange of gasses while at the same time facilitates the CTC-endothelium interaction. Pulmonary capillary endothelium is backed by a basement membrane between lung alveoli and pulmonary capillaries to allow gas exchange at the blood-air barrier (Weibel, 2015). This unique feature is now recognized to be of great pathophysiological significance. In summary, the lung vasculature is composed of metabolically active, functionally responsive cells, that interact with circulating substrates and regulate the composition of systemic arterial blood, affect target organ functions, and contribute to thrombosis, hemostasis, and immune reactions, as well as cancer metastasis. Therefore, a physiologically relevant microfluidics model that integrates the aforementioned key components of the lung microenvironment is essential for the understanding of breast cancer lung metastasis.

## The Liver Niche

Liver metastasis happens in about half of breast cancer patients (Narkhede et al., 2017). The liver is responsible for the metabolism of xenobiotics and drugs. For this reason, developing chemotherapeutics that can survive the liver microenvironment has been challenging. Several reasons contribute to the high incidence of breast cancer liver metastasis. First, liver sinusoid microvessels feature fenestrated endothelium to facilitate large molecule transport (Del Toro-Arreola et al., 2016), making it much leakier than endothelium at other organs. Second, the liver microenvironment provides essential cues for the extravasation of breast CTCs. This is largely due to the constant high pro-inflammatory chemokine and cytokine (e.g., IL-6, IL-8, and MCP-1) expression in the liver tissue which is regulated by the crosstalk between hepatocytes and non-parenchymal cells (endothelial cells, Kupffer cells, and leukocytes) (Wheeler et al., 2014). Last but not least, in order to perform its normal functionality, the liver microenvironment is immunosuppressed thus making it vulnerable for breast cancer colonization due to the lowered immune surveillance (Clark et al., 2016a).

Microfluidics approaches have been employed to mimic the hepatic niche using 3D functional tissue. A. Wells’ group achieved this by incorporating human hepatocytes, human non-parenchymal cells, and human breast cancer cells (MDA-MB-231 or MCF-7) in a commercially available (LiverChip) microscale bioreactor (Wheeler et al., 2013, 2014; Clark et al., 2016b). Using this microfluidic platform, Wheeler et al. (2014) showed that breast cancer cells successfully integrated with the hepatic tissue, however, a significant subset of cancer cells entered spontaneous dormancy (i.e., cease dividing but survive in a quiescent state) after ∼15 days of culture within the functional hepatic niche. The fraction of the cancer cells entering dormancy is associated with the presence of non-parenchymal cells which also altered the soluble factors (cytokines, chemokines, growth factors etc.) gradient within the liver niche (Wheeler et al., 2014). In a follow-up study by Clark et al. (2016b), the author, and colleagues investigated the effect of scaffold stiffness on the inflammatory phenotype in the liver niche using the same microfluidic platform. Their results revealed a positive correlation between scaffold stiffness and the aggressiveness of the metastasized breast cancer cells since the percentage of cancer cells entering dormancy was markedly increased in the hydrogel-supported tissue (softer) compared to polystyrene (stiffer) (Clark et al., 2016b). Overall, co-culturing breast cancer cells within the microfluidic liver niche comprised of hepatocytes and non-parenchymal cells provides a physiologically relevant platform to study most of the events of the metastatic cascade in the liver microenvironment. However, critical steps of breast cancer metastasis including circulating and extravasation were not modeled.

In addition to the soluble factors, EVs have been recognized as intercellular messengers between breast cancer cells and the PMN microenvironment at the distant organ. Recently, several research groups utilized microfluidics approaches to explore the role of breast cancer-derived EVs in the formation of liver PMN (Kim et al., 2020) and breast cancer liver metastasis organotropism (Tian et al., 2020). In the liver-on-chip microfluidic model developed by Kim, Cho and colleagues immortalized human liver sinusoidal endothelial cells were cocultured with immortalized human hepatocytes. EVs derived from breast cancer patients activated liver sinusoidal endothelial cells in the liver PMN, resulting in endothelial to mesenchymal transition and the destruction of the endothelial barrier function. This effect was not observed using EVs derived from healthy patients. In addition, the authors show that an upregulation of fibronectin, caused by cytokine TGFβ1 released from breast cancer-derived EVs, facilitated the adhesion of breast cancer cells to the liver microenvironment. As a result, TNBC patients with liver metastasis can attract more breast cancer cells to the liver niche as they produce EVs with higher TGFβ1 levels than do healthy donors or TNBC patients without liver metastasis (Kim et al., 2020). Compared to Kim’s system, Tian et al. (2020) took a slightly different approach. In their liver-kidney-on-a-chip microfluidic system, liver PMN was established by using precision-cut tissue slices that are harvested from Sprague-Dawley rats (Tian et al., 2020). A benefit of such an approach is that it retained the native chemokine secretion capability of the tissue and a natural chemokine gradient can be established in the microfluidic model. It is demonstrated that breast cancer EVs show strong liver tropism rather than kidney tropism on both the microfluidic and animal models and a CXCL12 mediated chemokine gradient that is unique to the liver PMN is responsible for the breast cancer EV organotropism. In summary, the breast cancer liver metastasis organotropism is due to the reciprocal interaction between primary breast tumor and the liver microenvironment. Breast tumor influence liver microenvironment via paracrine signaling (e.g., EVs) whereas the unique liver microenvironment (cytokine/cytokine profile, leaky endothelial cells, and suppressed immune surveillance) facilitates the extravasation and colonization of breast cancer cells in the liver.

## The Brain Niche

Breast cancer brain metastasis happens in about 10–15% of stage IV breast cancer patients (Breastcancer.org, 2020). At this stage, cancer has usually spread to multiple organs in the patients’ body. However, for about 17% of patients in this group (so in ∼2% of the stage IV breast cancer patients), the brain is the only organ of metastasis (Breastcancer.org, 2020). Modeling the BBB *in vitro* is crucial for the understanding of breast cancer brain metastasis. Efforts, including microfluidic approaches, have been directed into establishing a functional BBB. A summary of these studies can be found in the review article by Narkhede et al. (2017). The BBB is a highly specialized barrier that regulates the entry of molecules into the brain. Structurally, this complex barrier consists of endothelial cells supported by endothelial basement membrane forming the innermost layer, pericytes, supported by the parenchymal basement membrane, forming the middle layer, and astrocyte foot processes which cover more than 90% of the surface, forming the outer most layer of the BBB (Xu et al., 2019). BBB properties are primarily determined by tight and adherens junctions between the capillary endothelial cells (Stamatovic et al., 2008), and is regulated by the unique surrounding microenvironment (basement membrane, astrocytes, and pericytes) which controls the secretion of a variety of soluble factors that affect transport, signaling, angiogenesis and drug degradation, forming an enzymatic barrier (Eyal et al., 2009; Mehta et al., 2010; Wilhelm et al., 2013). The BBB is known to exclude nearly all molecules from entering the brain except those that are either small or lipophilic through membrane transporter proteins such as *P*-glycoprotein, multidrug-resistance proteins MDRP1-9, ABCG2 (the breast cancer resistance protein) and organic anion transporters (OATs) present on capillary endothelial cells (Deng et al., 2018; Gil-Martins et al., 2020). Normally, BBB also prevents the transmigration of blood cells and cancer cells, however, studies suggest that the defenses of the BBB can be disrupted in the presence of brain metastases (Brosnan and Anders, 2018).

**FIGURE 4:**
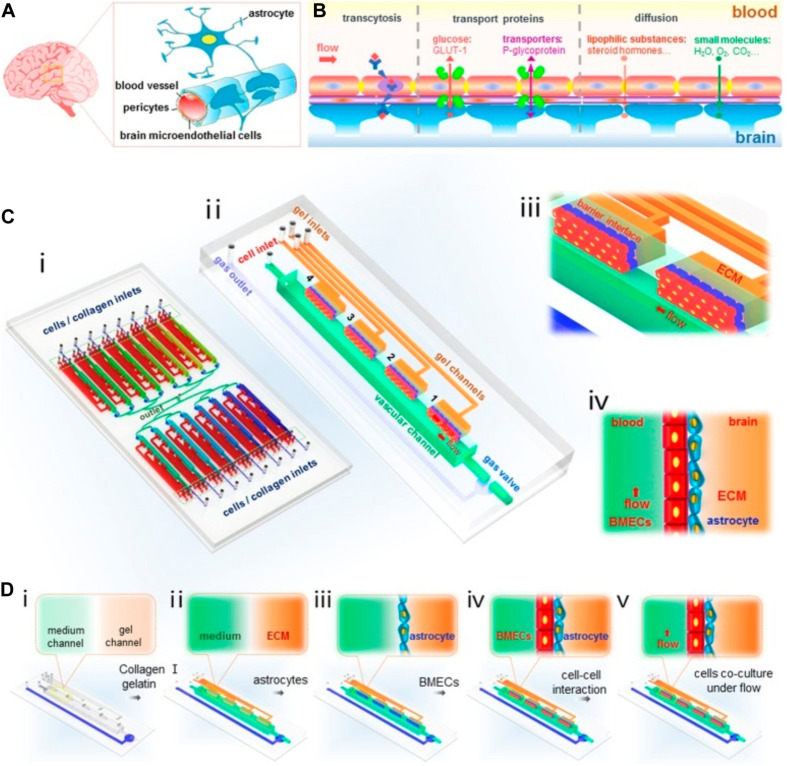
A Microfluidic design by Xu et al. (2016) to recapitulate BBB *in vitro* to probe breast cancer brain metastasis. A structural illustration of the *in vivo* BBB is provided in **(A,B)**. The design of the microfluidic device is provided in **(C)**. **(i)** 16 independent function units were connected via a microchannel network. **(ii)** Each unit consists of four uniform BBB regions, one vascular channel, one gas channel, one gas valve, and four gel channels which share the same waste outlet in the middle of the device. Enlarged view **(iii)** and side view **(iv)** of the barrier regions consisting of brain microvascular endothelial cells, astrocytes, and 3D ECM under flow. Procedures to establish the BBB were illustrated in **(D)**. Reproduced with permission (Xu et al., 2016). Licensed under CC BY-NC 4.0. https://creativecommons.org/licenses/by-nc/4.0/.

Breast cancer cells must extravasate through the BBB to establish metastasis in the brain tissue, and biochemical and physical interactions between metastatic breast cancer cells and the BBB affect the ability of cancer cells to transmigrate. Microfluidic BBB models have been created to explore the underlying mechanisms of this process. For example, Xu et al. (2016) constructed such an *in vitro* BBB model using co-cultured primary rate brain microvascular endothelial cells and astrocytes in PDMS microfluidic channels to examine the extravasation of breast cancer cells (Figure 4). Their findings suggest that the astrocytes in the BBB play a critical role in regulating the specific interactions between breast cancer cells and the endothelial cells, a result that is consistent with other *in vivo* and *in vitro* studies (Wang et al., 2013; Xing et al., 2013; Valiente et al., 2014; Chen Q. et al., 2016; Hohensee et al., 2017). To find out how exactly astrocytes regulate breast cancer metastasis to the brain, Shumakovich et al. (2017) established a microfluidic assay to study the transmigration of various breast cancers in response to astrocyte conditioned media (ACM). For this purpose, a microfluidic device was fabricated to have microchannels of varying widths (3 – 50 μm). The device contained four inlet wells. Cells were seeded in the bottommost well whereas a chemoattractant was added to the topmost well. The top channel contained serum alone which served as a positive control, serum-containing ACM alone, serum-containing ACM with serum as an additional chemoattractant, and serum-containing control media which served as a negative control. Their results suggest that although ACM does not serve as a chemoattractant for metastatic breast cancer cells, they alter cancer cell morphology and migration by modulating actin cytoskeleton organization, and this alteration can be reversed by inhibiting matrix metalloproteinases (MMPs) that were secreted by the astrocytes. Furthermore, comparing to direct treatment of ACM to the cancer cells, ACM treatment to the breast cancer ECM led to the most significant increase in cancer cell migration. Overall, although breast cancer cells of varying tumorigenic and metastatic potential respond differently to ACM treatment and the effects of ACM also depended on astrocytes’ activation state, it is clear that astrocyte-secreted factors can alter breast cancer migration, and this effect depends on the cells’ mechanical microenvironment.

Due to the presence of BBB, targeting brain metastasis has been challenging. In a study conducted by Oliver et al. (2019), machine learning algorithms were trained to predict the metastatic potential of aggressive TNBC cell lines and patient-derived xenografts (PDX) across the BBB (Oliver et al., 2019). This was attempted using a microfluidic blood-brain niche (μBBN) and confocal tomography for live-cell 3D imaging. Key motives for this research include developing a novel method for predicting a cancer cell’s potential to migrate across the BBB, leveraging artificial intelligence to identify migratory and proliferative phenotypes of cancer cells that are too subtle for manual identification, and ultimately lead to quicker, more accurate clinical decisions. Three main cell lines were compared: MCF-10A normal breast epithelial cell line, MDA-MB-231 TNBC cell line, and MDA-MB-231-BR, a brain-seeking subclone of MDA-MB-231. To model the BBB, an endothelial cell monolayer was formed using the HCMEC/D3 cells. MDA-MB-231-BR cells showed significant extravasation between 24 and 48 h. It was observed that the cytoskeletal plasticity of the cells enabled them to take on a more spherical shape during extravasation as opposed to the more elongated shape observed in leukocytes during this process. Before initiating colonization, the cells take on an elongated shape. Out of the several types of machine learning algorithms used to classify the metastatic potential of the cell lines, neural networks (accuracy 0.871 for cell lines and 0.881 for PDX), Adaboost (accuracy 0.876 for cell lines and 0.888 for PDX), and the random forest (accuracy 0.874 for cell lines and 0.881 for PDX) showed the best performance. The algorithms were also used to classify data for brain metastatic PDX cells derived from various primary sites and primary breast cancer PDX cells which were used as non-brain metastatic controls. Future studies will need to better recapitulate the BBB in microfluidic devices through the introduction of fluid flow and additional cell types.

A key benefit of applying AI techniques in biomimetic microfluidic experiments is to extract features from cancer cell phenotypes and then use these features as inputs into machine learning models which can classify the identity of different cell types or disease stages or predict the likelihood that a cancer cell will metastasize. Although biomimetic microfluidic platforms do not perfectly mimic their *in vivo* counterparts, they enable the feasible collection of large amounts of data which is necessary for such AI applications. Even a single time-lapse experiment can easily generate on the order of 100 gigabytes of data (Riordon et al., 2019). Furthermore, Microfluidic devices enable features to be extracted from dynamic biomarkers present in live cells as opposed to being limited to static biomarkers found in fixed cells (Manak et al., 2018). For example, the previously mentioned study conducted by Oliver et al. (2019) utilized a variety of common classification algorithms such as Neural Networks, Adaboost, and Random Forests to predict the potential of cells from normal and cancerous breast cell lines and PDX to migrate across the BBB (Oliver et al., 2019). Currently, there is a lack of other such studies that apply AI techniques to make predictions based on microfluidic platforms mimicking the breast cancer TME. However, recent research in related areas has applied machine learning more generally to microfluidic platforms. For example, Wang et al. (2018) developed a single-channel microfluidic device and used polarization microscopy to classify CAFs and two different non-small cell lung cancer cell lines, A549 and H322, via logistic regression and gradient descent with regularization. Their classification algorithm achieved 66.7% accuracy. On-chip molecular biomarker screening enables multiple molecular biomarkers derived from cellular or vesicular proteins and different kinds of nucleic acids to be quantified in terms of concentration of relative expression (Molinski et al., 2020). This then results in large datasets with high dimensionality which is suitable for the application of machine learning to find patterns in data or perform classification tasks. Exosomes contain vast amounts of proteomic and genomic information. However, their small size makes it infeasible for most microfluidic platforms to isolate exosomes. Ko et al. (2017) created a device containing millions of nanoscale immunomagnetic components which sort exosomes in parallel. From isolated exosomes, features derived from multiple RNA biomarkers were fed into a linear discriminant analysis (LDA) algorithm which was able to correctly differentiate murine and clinical cohorts with pancreatic cancer and healthy controls.

## Conclusion

Microfluidic approaches aim to reconstitute functional units of organs that cannot be modeled easily in traditional cell culture or animal studies. Table 1 summarizes the most up-to-date use of microfluidic-based devices for recapitulating breast cancer metastatic processes. In recent years, microfluidics models utilizing innovative biomaterials and state-of-the-art microengineering technologies have shown great potential in our effort of mechanistic understanding of breast cancer metastasis cascade by providing 3D constructs that can mimic *in vivo* cellular microenvironment and the ability to visualize and monitor cellular interactions in real-time. However, its application is also restricted by low cell number, small operating volume, a limited selection of substrates, and tunability of culture conditions (Halldorsson et al., 2015). Furthermore, while excellent advances have been made, much like other *in vitro* methods, microfluidics approaches often suffer from bias and lack of translational relevance on account of their heavily artificial nature. There is, therefore, a critical and urgent need for the development of platforms that focus on increasing the physiological relevance of these models, including, but not limited to, using organ-specific primary endothelial cells, tissue cells, and PDX breast cancer cells to replace the commonly used HUVECS and commercially available immortalized cancer cell lines; incorporating EVs and immune cells to better recapitulate the complex paracrine signaling in regulating breast cancer metastasis and organotropism; large scale, integrated on-chip proteomics/genomics analysis and proper validation of the results using relevant *in vivo* animal studies. In summary, future microfluidics models should provide a reliable foundation for the generation of AI-based *in silico* models for the prediction of the metastatic potential of patient samples, effectiveness and efficacy of novel therapeutics, and specific treatment regimens for personalized medicine.

**TABLE 1:** Summary of microfluidic devices used in studies of breast cancer metastasis.

References	Cells used	Device properties	Metastatic niche	Remarks
Yankaskas et al., 2019	MDA-MB-231 and Patient-Derived Xenografts (PDX)	-Microfluidic Assay for the quantification of Invasion -Comprised of parallel Y shaped microchannels	Invasion	-Comparison showed migratory cells correlated with their metastatic potential -Device has the potential to be developed into metastatic prediction assay.
Gioiella et al., 2016	Epithelial Breast Cancer Cells (MCF7), stroma (fibroblast- assembled ECM)	-PDMS based device with barrier region in between cells -Co-culture of MCF7 and stroma in a single device	Invasion	-Higher degree of similarity observed when compared to *in vivo* models.
Blaha et al., 2017	MDA-MB-231 and Human Umbilical Vein Endothelial cells (HUVECs)	Microfluidic chamber having 3D collagen matrix constructs -Co-culture of MDA-MB-231 and HUVEC in the collagen matrix	Invasion	-Cancer cell invasion significantly increased in the presence of HUVEC cells
Truong et al., 2019	Breast cancer (SUM-159) and Cancer Associated Fibroblasts (CAFs)	-Microfluidic 3D organotypic model -PDMS based device for co-culture of cells to recapitulate the breast tumor microenvironment	Invasion	-Helped cancer-stroma communication -Increased cancer cell migration speed -Transcriptome analysis revealed novel molecular targets associated with breast cancer invasion
Cui et al., 2017	Primary human vascular endothelial cells and MDA-MB-231 cells	-Microfluidic device developed using photolithography of SU-8 photoresist -Consisted two flow layers along with a central porous membrane (4 mm*4 mm) -Bottom layer consisted of multiple microchamber for cell collecting	Intravasation	-Quantified transendothelial migration of breast cancer cells under different stress levels, i.e., 2.5 dyne/cm^2^ and 10 dyne/cm^2^ -More breast cancer cells migrated through the endothelial layer for low shear stress as compared to the high-stress level
Nagaraju et al., 2018	HUVECs, MDA-MB-231, and MCF7	-Microfluidic device consisting of three layers: inner layer for tumor cells, central for stroma, and outer layer for vasculature as well as surrounding media channels -Tumor and stroma layer consisted of collagen as a major protein whereas fibrin was selected for the vascular layer	Intravasation	-Higher number of cancer cells invaded the stromal region in presence of endothelial vascular networks -Different morphologies were observed for cancer cells in the presence or absence of endothelial cells in the vascular network -Presence of cancer cells make endothelial networks to be leakier and more permeable - Endothelial cells are a key source for leading the intravasation process
Shirure et al., 2018	Normal human lung fibroblasts (NHLFs), Endothelial colony-forming cell-derived endothelial cells (ECFC-ECs), MDA-MB-231, MCF-7, and colorectal cancer cell line (Caco-2)	-Consisted of three distinct tissue layers parallel to each other -Communication between the layers is via microporous walls -Central layer was for vascular networks while side layers for cells loading	Intravasation	-The designed device was able to biologically mimic a relevant tumor microenvironment between the arterial end of capillary and tumor -Device can culture wide variety of cancer cell -Primary tumor organoids prepared were workable for several weeks
Chen M. B. et al., 2016	HUVEC (Human Umbilical Vein Endothelial Cells), NHLF (Normal human lung fibroblasts), MDA-MB-231, A-375 MA2, and 4T1 cells	-Microfluidic based device with microvascular networks and three independent hydrogel regions separated by media channels in between. -HUVECs (human umbilical vein endothelial cells) and NHLFs (Normal human lung fibroblasts) were used for creating vascular networks	Extravasation	-B1 integrin expression was an important aspect for transendothelial migration of breast cancer cells along with stabilizing protrusions and contacting the basement membrane -Further studies are needed to learn more about the role of B1 integrin.
Chen et al., 2017	HUVECs and NHLFs	-PDMS based device for microvasculature extravasation examine	Extravasation	-Tumor cells extravasation from smaller vessels showed greater physiological relevance than traditional models -All processes like tumor cells interacting and invading endothelial basement membrane tracked via immunofluorescent techniques
Song et al., 2018	HUVECs, NHLF MCF-10A, MCF-7, and MDA-MB-231	-Device fabricated using PDMS (polydimethylsiloxane) using standard photolithography -Device consisted of a central gel layer and two media channels	Extravasation	-Cells were exposed to two different conditions, i.e., hypoxia and normoxic state -HIFs: markers for hypoxia -Knockdown of HIF-1α in hypoxic tumor decreased the extravasation rate of all cancer cells lined tested
Boussommier-Calleja et al., 2019	Cytoplasm-labeled GFP-endothelial cells (HUVECs), NHLF, Monocytes, MDA-MB-231, and MDA-MB-435	-PDMS based microfluidic device composed of five channels connecting two cell media reservoirs compartment -Central compartment filled with a cell-hydrogel mixture	Extravasation	-Monocytes affects cancer cell extravasation and has a key role in the metastatic process -Replicated phenomena seen *in vivo* as well as the discovered undefined role of monocytes in the tumor microenvironment -This device promises a powerful model for anti-cancer drug therapy in future
Bersini et al., 2014	Bone marrow-derived human mesenchymal stem cells (hBM-MSCs), Red fluorescent protein (RFP)-HUVECs, MDA-MB-231	-Microfluidic device consisted of three media channels and four independent channels for gel -Device fabricated using PDMS -8 gel regions having to interface with central media channel were adopted for cell interactions study	Breast cancer bone metastasis	-CXCR2 and CXCL5 are the major events resulting in extravasation and the migration rate of cancer cells -Inhibition of those agents can serve as anti-cancer drug therapy and shows the therapeutic potential
Jeon et al., 2015	hBM-MSCs, GFP-HUVECs, osteo-differentiated hBM-MSCs, MCF-10A, MDA-MB-231	-PDMS based device with central hydrogel compartment along with lateral media channels -Cover glass bonded to PDMS and ports created by biopsy punches	Bone Metastasis and Extravasation	-Cancer cell extravasation rates significantly higher in the bone microenvironment -A3 adenosine receptor showed potential in the prevention of breast cancer bone extravasation -Presence of flow condition showed a favorable environment for cancer cell migration into the surrounding matrix
Mei et al., 2019	MLO-Y4 cells, RAW264.7 cell line, HUVECs, MDA-MB-231	-Microfluidic device consisting of osteocyte channel, lumen channel, and side channels fabricated using PDMS -Microfluidic chip were arrayed for increasing throughput	Breast cancer bone metastasis	-Oscillatory fluid flow was induced that was relevant to the physiological model -Flow condition reduced the number of extravasated cells as well as the distance traveled by them when compared to non-flow conditions
Kong et al., 2016	HUVECs, MDA-MB-231, MCF-7, and ACC-M	-Device consisted of four layers: one glass substrate layer, two PDMS membrane, and one porous membrane -Porous membrane in between PDMS layers -four parallel and branched microchannels for creating vasculature on top of PDMS layers.	Breast cancer lung metastasis	-MDA-MB-231 showed greater lung metastasis potential among all cancer cell lines tested in the microfluidic model developed -When compared with the animal model, the microfluidic model showed physiological similarity
Kim et al., 2020	Liver epithelial THLE-2 cells, Primary liver fibroblasts (LFs), Human liver sinusoidal endothelial cells (LSECs), liver hepatocytes, MCF-7, MDA cell lines, and MCF-10A	-Platform consisted of the top layer, bottom layer, and middle layer -Middle layer made of thin porous PDMS membrane interfaced with top and bottom layers	Breast cancer liver metastasis	-This device enabled the recapitulation of the human liver microenvironment consisting of distinct types of liver cells -Breast cancer cell adhesion increased by breast cancer-derived extracellular vesicles EVs in the liver niche
Tian et al., 2020	HUVECs, MCF-7, MDA-MB-231	-Device consisted of two tissue chambers and two media channels -Media channels joined to inlet and outlet on each end -Porous membrane in between two PDMS layers	Breast cancer liver metastasis	-Precision cut tissue slices (PTS)-based liver–kidney on a chip model was developed
Xu et al., 2016	Astrocytes, primary rat BMECs, A549, MDA-MB-231, M624, and BEL-7402	-Device had 16 independent units connected by a microchannel network -Each unit had four uniforms BBB (Blood Brain Barrier) regions and shared the same outlet in the middle of the chip	Blood-brain barrier model and extravasation	-Developed device effectively replicated BBB in normal and diseased conditions -Astrocytes in BBB plays a vital role in interactions between endothelial cells and cancer cells -Results were consistent with other *in vivo* and *in vitro* studies
Oliver et al., 2019	Astrocytes, HCMEC/D3 endothelial cells cancer cells, MCF-10A, MDA-MB-231, MDA-MB-231-BR, and brain seeking subclone of MDA-MB-231	-Machine learning algorithms trained for prediction of metastatic potential of breast cancer cells across BBB regions -Microfluidic device composed of two chambers separated by a porous membrane -Fabricated using PDMS	Extravasation of cancer cells into brain metastatic niche	-Neural networks, Adaboost and Random forest showed the best results -MDA-MB-231-BR showed significant extravasation compared to other cells -Future studies involving fluid flow and the addition of cells would help better recapitulation of BBB *in vitro*.

## Author Contributions

IS, NG, and FF prepared the manuscript. VK edited the manuscript. AT and YT developed the ideas and edited the manuscript. All the authors contributed to the article and approved the submitted version.

## Conflict of Interest

VK is employed by company Eurofins Lancaster Laboratories. The remaining authors declare that the research was conducted in the absence of any commercial or financial relationships that could be construed as a potential conflict of interest.
